# Staying true to yourself: mechanisms of DNA methylation maintenance in mammals

**DOI:** 10.1093/nar/gkaa1154

**Published:** 2020-12-09

**Authors:** Nataliya Petryk, Sebastian Bultmann, Till Bartke, Pierre-Antoine Defossez

**Affiliations:** Epigenetics and Cell Fate Centre, UMR7216 CNRS, Université de Paris, F-75013 Paris, France; Department of Biology II, Human Biology and BioImaging, Ludwig-Maximilians-Universität München, 80539 Munich, Germany; Institute of Functional Epigenetics, Helmholtz Zentrum München, 85764 Neuherberg, Germany; Epigenetics and Cell Fate Centre, UMR7216 CNRS, Université de Paris, F-75013 Paris, France

## Abstract

DNA methylation is essential to development and cellular physiology in mammals. Faulty DNA methylation is frequently observed in human diseases like cancer and neurological disorders. Molecularly, this epigenetic mark is linked to other chromatin modifications and it regulates key genomic processes, including transcription and splicing. Each round of DNA replication generates two hemi-methylated copies of the genome. These must be converted back to symmetrically methylated DNA before the next S-phase, or the mark will fade away; therefore the maintenance of DNA methylation is essential. Mechanistically, the maintenance of this epigenetic modification takes place during and after DNA replication, and occurs within the very dynamic context of chromatin re-assembly. Here, we review recent discoveries and unresolved questions regarding the mechanisms, dynamics and fidelity of DNA methylation maintenance in mammals. We also discuss how it could be regulated in normal development and misregulated in disease.

## INTRODUCTION

### CpG methylation: an essential epigenetic mark in mammals

DNA methylation is a covalent modification of the genome with a long evolutionary history: it exists in all kingdoms of life from bacteria and archaebacteria to eukaryotic organisms. Part or all of the methylation machinery has been lost independently in multiple phyla (1–4), for instance there is no CpG methylation in common model organisms such as *Saccharomyces cerevisiae*, *Caenorhabditis elegans* or *Drosophila melanogaster*, yet among eukaryotes, many species display DNA methylation (5–7). In mammals, which will be the focus of this review, DNA methylation is essential for embryonic development and cellular function (8–10).

In mammals, the most abundant form of methylated DNA is 5-methyl-cytosine (5mC). Non-CpG methylation is detected in neurons and other cells (11,12) however the main context in which cytosine methylation occurs is within CpG dinucleotides, which are palindromic. About 80% of CpGs in the genome are methylated in a typical mammalian cell and the methylation is mostly symmetrical (i.e. the methyl group is present on both strands). The pattern of DNA methylation varies across the genome: the level of CpG methylation is high in repeated elements, intergenic regions and gene bodies, while the vast majority of CpG islands, i.e. regions with high CpG density, escape DNA methylation (13). Molecularly, CpG methylation is integrated within the broader functional network of chromatin modifications (14,15). For instance, the histone mark H3K9me3 and CpG methylation frequently co-occur and reinforce one another, while DNA methylation and H3K4me3 exclude one another (16). Similarly, DNA methylation and the histone variant H2A.Z are also mutually antagonistic (4,17).

CpG methylation has two interdependent roles: the mark regulates gene expression and ensures genome stability (5,10,13). Indeed, the methylation of CpG island promoters locks them in a repressed state, which is key to ensuring the proper expression pattern of germline-specific and pluripotency-associated genes, and in regulating promoter choice and gene expression during development. CpG methylation is also crucial for keeping repetitive DNA elements such as retrotransposons tightly repressed and preventing them from destabilizing the genome and perturbing the cellular transcriptome (18–20). DNA methylation is also instrumental in maintaining the proper heterochromatic structure of centromeres, which is essential for accurate chromosome segregation (21). Finally, DNA methylation in the body of genes is positively correlated with gene expression and has been proposed to suppress spurious initiation of transcription (22,23). In addition to regulating transcription, DNA methylation also influences splicing (24–26) and alternative transcript polyadenylation (27).

Different cell types have different methylomes, which are instructive for cell identity (28,29). For instance, it has been clearly shown that one of the limiting steps of cellular reprogramming is the erasure of DNA methylation (30). A progressive deterioration of DNA methylation patterns occurs during aging, which is characterized by a global hypomethylation throughout the genome combined with focal hypermethylation (31,32). Similar defects are seen in cancer cells, where they correlate with deregulated gene expression, loss of silencing of repeated elements, genomic instability, and perturbation of cellular identity (33–36).

As the pattern of DNA methylation is dynamic during development and cellular life (9), the machinery that maintains DNA methylation must be able to faithfully reproduce the DNA methylation patterns that define cellular identity and maintain genomic integrity, while also being flexible enough to allow the programmed changes to take place.

### Hemi-methylated DNA is formed during DNA replication. The maintenance enzyme DNMT1 requires UHRF1 to function

The DNA replication machinery incorporates the available dCTP, which is normally unmethylated, thus newly-replicated DNA is hemi-methylated, with methyl groups only on the parental strands. Therefore, the faithful propagation of DNA methylation patterns through DNA replication requires the conversion of hemi-methylated sites back to symmetrically methylated molecules, to prevent loss of the mark.

Two remarkably prescient papers postulated the existence of such a ‘maintenance’ DNA methyltransferase as early as 1975 (37,38); their prediction was validated when Bestor and Ingram cloned the corresponding enzyme, DNMT1 (39). Mouse embryos bearing an inactivating point mutation in the catalytic domain of DNMT1 die during development, establishing that the catalytic activity of DNMT1 is vital (40,41).

Mammalian cells can also express two other DNA methyltransferases, DNMT3A and DNMT3B. These enzymes have a key ‘de novo’ methylation activity, establishing new patterns of cytosine methylation on DNA that was previously unmodified (10,22,42–45). In addition, in cell types where they are expressed, these enzymes contribute to DNA methylation maintenance, in part because they oppose active demethylation by the TET enzymes (46–48). However, as the bulk of the maintenance activity is carried out by DNMT1 (8), it will be the main focus from here on, and we will return to the ‘de novo’ methyltransferases again only in the discussion.

Early models postulated that DNMT1 might have a simple mode of action, with straightforward recruitment to hemi-methylated CpGs. Over the years, it has become apparent that the mechanisms underlying DNMT1 action are considerably more complex than initially thought. Not only has it become clear that the activity of DNMT1 is tightly regulated by intramolecular events (49,50), it also emerged that the enzyme requires additional factors, including the protein UHRF1 (51,52), which is itself intricately controlled (53), as we will develop later on. One likely cause for these complex regulations is that DNA methylation maintenance has to be orchestrated not on naked DNA, but in the context of chromatin.

### DNA methylation maintenance occurs in the context of chromatin replication

DNA methylation maintenance is coupled in space and time with DNA replication, which itself occurs on a chromatinized DNA template. The chromatin template, however, poses a specific challenge during replication. First, nucleosomes are an obvious impediment to the DNA replication machinery, and they need to be disassembled ahead of the moving forks. Second, nucleosomes also need to be re-assembled after DNA synthesis. As there are now two DNA helices instead of just one, twice the number of histones is now needed to package the DNA, and the delivery and loading of new histones onto the newly duplicated DNA must be coordinated with the recycling of the old histones. Third, nucleosomes carry a rich complement of information in the form of specific post-translational modifications of the histones and in the nature of the histones that they contain, such as canonical histones versus histone variants (54). New histones lack these modifications. Hence, this information must somehow be transmitted to both daughter strands so as to ensure epigenetic inheritance through replication (55). A further complication in preserving these unique chromatin signatures is linked to the directionality of DNA synthesis: the leading and lagging strands employ distinct mechanisms to achieve the synthesis of the new DNA, requiring different proteins and enzymes, whose activities are coordinated in the replisome (56).

### Some key questions remain unanswered

Much ground has been covered since the discovery of DNMT1, and many aspects of DNA methylation maintenance have been clarified. Nevertheless, some old questions remain and some new questions have arisen. Based on the most recent findings in the field, we will discuss:

1. How does DNA methylation maintenance take place in the context of chromatin replication? In other words, how is DNA methylation maintenance coordinated with DNA synthesis, histone loading and recycling, and the re-establishment and maturation of chromatin? What steps are subject to regulation, allowing for functional plasticity during differentiation and development, but also leading to potential misregulations in disease? Do DNA methylation maintenance processes have an impact on DNA repair or vice versa?
2. What is the speed and fidelity of the maintenance process? Which events are co-replicative, which occur post-replication, and what is the difference? Are the methylation kinetics similar in different parts of the genome or in different chromatin types (early vs. late replicating, active versus silent chromatin)? Where does the methylation drift observed in older cells come from?
3. Are there differences between leading and lagging strand DNA methylation maintenance, and can this have functional consequences?

### Section 1. Coordinating DNA methylation with DNA replication and chromatin assembly

**Figure 1.**
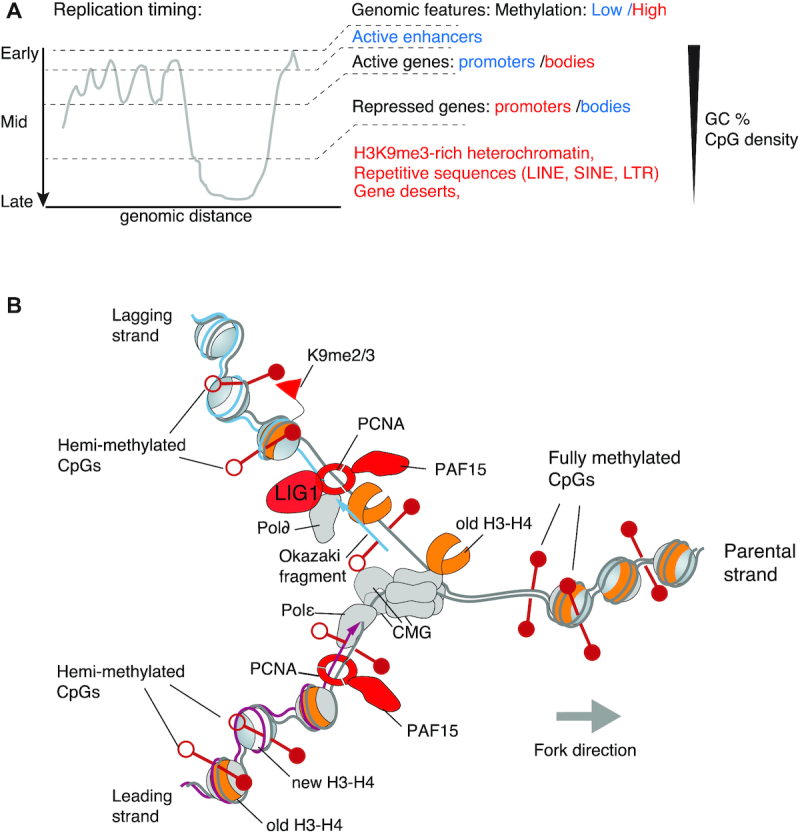
The landscape and machinery of chromatin replication. (**A**) Replication timing along a segment of mammalian chromosome. Horizontal axis: genomic distance along the segment. Vertical axis: time at which the region is replicated during S-phase. The functionally different elements of the genome are replicated at distinct times, for instance enhancers replicate early and heterochromatin replicates late. We also indicate the typical CpG-richness of these elements (triangle at the right), and whether these CpGs are mostly unmethylated (in blue) or methylated (in red). (**B**) DNA methylation maintenance in the context of DNA replication and chromatin assembly. This scheme is simplified and only presents the actors mentioned in the text. Parental DNA strands are in grey, leading DNA strand in magenta, lagging DNA strand in blue. CpG dinucleotides are represented by lollipops, which are filled in white when unmethylated, and in red when methylated. The nucleosomes are shown as balls, with ‘old’ H3–H4 in orange, and new H3–H4 in white. Some of the old H3 contain H3K9me3 modifications (red flags), whereas the newly synthesized H3 do not. The DNA replication machinery generates hemimethylated CpGs.

It is outside the scope of this article to review DNA replication itself, yet we need to introduce a few fundamental concepts and actors that are relevant to our discussion:

1. The replication of large mammalian chromosomes requires multiple replication origins, i.e. sites where DNA synthesis starts (57,58). DNA replication origins are licensed in the G1 phase of the cell cycle by loading divergently oriented pairs of the replicative helicases MCM2–7 (59); those are initially inactive and become activated only in S-phase when origins fire and DNA synthesis is initiated (60).
2. The replication of distinct genomic regions can occur at different times within S-phase (Figure 1A): some replication origins fire early and some later in S-phase (61). This timing is cell-type specific; it depends on the local transcriptional program, chromatin accessibility, and 3D-genome organization (62).
3. Upon origin activation, the paired MCM helicases separate and through binding of additional factors are converted into active helicases called CMG (Cdc45-MCM2–7-GINS, Figure 1B), which melt the parental DNA helix (63). The leading strand is synthesized continuously by DNA polymerase Epsilon (Polϵ); the lagging strand is synthesized discontinuously, in the opposite direction, in short Okazaki fragments. Each Okazaki fragment is initiated by the DNA polymerase Alpha-primase complex, synthesized by DNA polymerase Delta (Polδ), processed, and then ligated by DNA Ligase 1 (56). When two convergent neighboring replication forks merge, the replication of the locus terminates (64).
4. PCNA is a core regulatory component of the eukaryotic replisome. PCNA promotes processive DNA synthesis but is also an interaction hub and acts as a functional switch for many factors of DNA replication, DNA repair and chromatin assembly (65). PCNA is essential for synthesis of both the leading and lagging strands; on the leading strand PCNA is loaded only once during the initiation step (and, if necessary, during fork restart), while on the lagging strand PCNA is loaded during each Okazaki fragment initiation and coordinates the enzymes of Okazaki fragment processing (56).

Concomitantly to DNA replication, chromatin is also replicated (Figure 1B). This phenomenon has been studied in depth, and many excellent reviews exist (55,66–68). The following notions are essential for our discussion of how DNA maintenance methylation is linked to DNA replication:

1. Nucleosomes are disassembled ahead of the fork and broken up into H3–H4 tetramers and H2A–H2B dimers. The nucleosomes are immediately reassembled behind the fork in the inverted order: the initial deposition of an H3–H4 tetramer is followed by adding two H2A–H2B dimers (55,66,67). This process is carried out by histone chaperones, and it is tightly coordinated with DNA synthesis, often by direct physical contacts between the two machineries (55,69,70).
2. The newly-replicated chromatin consists of recycled ‘old’ histones, and an equal amount of ‘new’ naïve histones. During replication the patterns of locus-specific modifications are preserved, as the old histones, bearing functionally important marks, are transmitted to both daughter chromatin strands (55). How this occurs is known for old H3-H4 tetramers: on the leading strand, their recycling is mediated by DNA Polϵ (71,72), and on the lagging strand by MCM2 (73,74). Due to the balanced activity of these two pathways, the original patterns of locus-specific histone marks are on average reproduced nearly symmetrically on the new genome copies (55). However, the old histones are interspersed with new histones, which must progressively gain the appropriate marks. This maturation process is uncoupled from DNA replication and can take much longer than replication itself, extending into G2 or even the following G1 (75–77). Importantly, different histone marks have different maturation kinetics, affecting the speed with which different chromatin domains are re-established during the cell cycle (76,78).

### Section 2. The main actors of DNA methylation maintenance: DNMT1 and UHRF1

**Figure 2.**
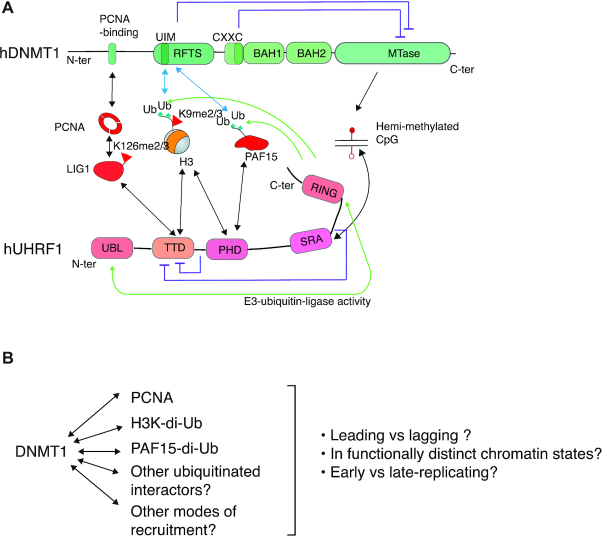
DNMT1 and UHRF1, key actors of DNA methylation maintenance. (**A**) Domain architecture and interactors of DNMT1 (top) and UHRF1 (bottom). Bidirectional black arrows indicate interactions. Green arrows show enzymatic modification. Lilac arrows denote inhibitory interactions. (**B**) Several pathways have been shown to permit DNMT1 recruitment. Additional pathways may remain to be discovered. It is not yet clear which modes of recruitment predominate in different situations.

DNMT1 is a large enzyme with many accessory regions besides the catalytic domain (50,79) (Figure 2A). It was realized early on that DNMT1 interacts with the replication protein PCNA, suggesting a targeted recruitment mechanism to replicating regions and a coupling of DNA methylation to DNA replication (80). However, further experiments established that the DNMT1/PCNA interaction is actually dispensable for cells to maintain their global DNA methylation level (81,82), suggesting either that this recruitment to forks is not followed by DNA methylation, or that parallel pathways of DNMT1 recruitment exist. The discovery of some of these mechanisms took a decade and will be described now.

The DNMT1 enzyme is processive, yet intramolecularly inhibited, both by a CXXC-type Zinc finger domain (which binds unmethylated CpG and limits the de novo activity), and by the larger RFTS domain (for Replication Foci Targeting Sequence) (50,83). The RFTS has a ubiquitin-interaction motif, and its inhibitory effect is lifted when it engages histone H3 bearing mono-ubiquitin at lysines 14 and 18 (84–86). The deubiquitinating enzyme USP7 removes ubiquitin from H3, presumably freeing DNMT1 to act on further loci (87); besides this positive role, USP7 seems to have an inhibitory role on maintenance, by limiting the amount of ubiquitinated histones available to activate DNMT1 (88). However, some controversy exists as to the involvement of USP7 in DNA methylation maintenance (89); the reason for the contrasting results is unclear but could possibly have its source in different cellular models. Fascinatingly, a histone H3-like motif is present on the PCNA-interacting factor PAF15. Like H3, it is ubiquitinated on two close lysines, creating a site that binds and allosterically activates DNMT1, which is important for DNA methylation (90).

Besides this body of work on allosteric regulation of DNMT1 activity, several studies have shown that post-translational modifications of the enzyme affect its stability and/or activity (31,91–96). These modifications determine where and when DNMT1 can be active and could have a direct impact on DNA methylation maintenance.

The second factor that is indispensable for maintenance DNA methylation is UHRF1 (51–53). UHRF1 is the E3 ubiquitin ligase that ubiquitinates both H3 and PAF15 (Figure 2A), resulting in DNMT1 recruitment and methylation of hemi-methylated CpGs generated during DNA synthesis (90,97,98). Its E3 ubiquitin ligase activity is carried by a C-terminal RING finger, and is regulated by an N-terminal Ubiquitin-like domain (UBL), which contacts the E2 enzyme and might be involved in directing mono- rather than poly-ubiquitination to the substrate (99,100).

In addition to this enzymatic activity, UHRF1 has three well-characterized modules that bind proteins or DNA (Figure 2). Next to the UBL domain are a Tandem Tudor Domain (TTD) and a Plant Homeo Domain (PHD). The PHD domain binds the sequence ARTK at the very N-terminus of H3, (101) and also binds a very similar peptide, VRTK, at the very N-terminus of PAF15 (90). The PHD domain cooperates with the TTD to bind H3K9me3 (102,103). Furthermore, the TTD also has non-histone ligands. First, it binds two linkers within UHRF1 itself, leading to intramolecular inhibition (104–108). Second, it can bind a methylated histone-like motif in the replication protein LIG1 (109,110). The TTD targets are mutually exclusive: binding to the intramolecular linkers or to methylated LIG1 precludes binding to histones. The TTD, with this variety of interactors, would seem to play a central part in the function of UHRF1. However, despite this seemingly critical function of the TTD, in mouse ES cells (111,112), and in human cancer lines (113), a mutant version of UHRF1 with an inactivated TTD can almost fully substitute for the wild-type protein to ensure steady-state DNA methylation levels. The last structural domain of UHRF1 is the SRA (SET and RING finger-associated), which binds hemi-methylated CpGs and also, with less affinity, fully-methylated or even unmethylated CpGs (53,114); this activity is essential for DNA methylation maintenance in cells (111,113).

Similar to DNMT1, UHRF1 can be regulated by post-translational modifications that modify its stability (95,115,116), and also undergoes complex allosteric regulations. For instance, engagement of hemi-methylated DNA by the SRA activates histone binding and ubiquitination (117–119). Binding to USP7 displaces a linker and frees up the TTD to interact with its ligands (120). Binding of LIG1K126 also opens up an otherwise compact structure, though the effect on DNA binding or RING finger activity are undetermined (106). Furthermore, post-translational modifications of UHRF1 also control its activity and stability, and provide additional regulatory inputs (95,107,116,121–124).

In summary, the activities of UHRF1 and DNMT1 are modulated by a number of intricate regulations. A major challenge is to place those events in space and time, relative to the other processes taking place during chromatin replication.

### Section 3. DNA methylation maintenance in the context of chromatin replication

The replisomes are complex molecular assemblies, containing all the actors described above: CMG helicases, polymerases, PCNA and its many interactors… They coordinate DNA unwinding, nucleosome disassembly and reassembly, and DNA synthesis. How does DNA methylation maintenance occur in this context?

A number of actors and events have been convincingly described, as stated above. However, inserting them in the broader context of chromatin replication is often complex, as the order of events is not clearly established and a number of steps are probably still unknown. For instance, how is UHRF1 released from hemi-methylated CpGs so that DNMT1 can gain access to them? If DNMT1 is indeed activated by ubiquitinated H3, then it must be necessarily be recruited after nucleosomes have been reassembled. But then again, DNMT1 cannot act on nucleosomal DNA (47,125), it therefore either needs to act before nucleosomes are assembled, or the action of a nucleosome remodeler is required for DNMT1 to gain access to the underlying sequence.

Replication necessarily increases the accessibility of the chromatin and leads to the incorporation of new histones, which are acetylated, and therefore promote a more open structure until they become deacetylated. For about 20 min after replication, chromatin maintains this higher accessibility (66), and it is likely that this time window promotes maintenance of DNA methylation. A supporting argument is that the removal of a chromatin assembly factor, CAF1, speeds up methylation maintenance (126). Interestingly, UHRF1 decorates H3 tails with bulky ubiquitin modifications, which potentially also destabilize the nucleosomal organization of chromatin and might facilitate the access of DNMT1 to the hemi-methylated DNA.

While part of the maintenance activity may take place on relatively open chromatin, it is clear that some remodeling activity is also required for the maintenance to be complete. The helicase LSH is essential to shift nucleosomes so that DNA can be de novo methylated (127,128), and it has recently been shown to play the same role for maintenance methylation (126). We note that fusing DNMT1 directly to PCNA can partially bypass the requirement for UHRF1 (111). Where and when this chimeric DNMT1 acts is unknown.

One difficulty in understanding how DNA methylation maintenance fits within the broader picture of chromatin replication is a relative dearth of time-resolved data. Fortunately, recent publications have started to fill this gap, as we will now discuss.

### Section 4. Speed, fidelity and symmetry of the maintenance

The replication fork proceeds at approximately 1 kb/minute (129). Nucleosomes are ∼200 bp apart in mammalian cells (130,131), and there are ∼20 million CpG dinucleotides in the 3-megabase human or the mouse genome, of which 80% are methylated on average (5). Therefore, a rough estimation would be that each nucleosomal unit contains one methylated CpG, and that five such repeats are replicated every minute by one given fork.

Early enzymological studies suggested that the replication fork was generating hemi-methylated DNA faster than DNMT1 could possibly methylate it (132). In parallel, microscopy suggested that DNMT1 was associated with chromatin through S, G2 and M, suggesting that indeed, DNMT1 was catching up on maintenance long after DNA replication had been completed (133). However, the speed at which DNA methylation is re-established on newly replicated DNA has been a matter of debate.

**Figure 3.**
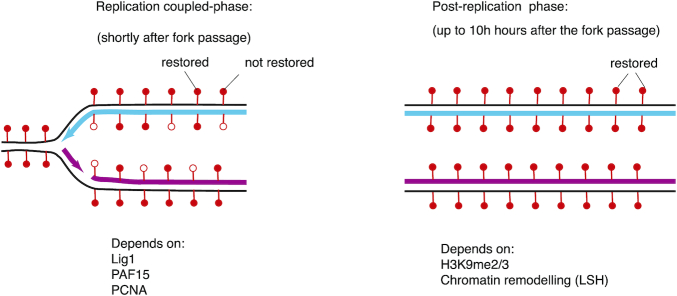
Different kinetic phases of DNA methylation maintenance. The kinetics of DNA methylation maintenance combine a replication-coupled component, operating within minutes of DNA replication (left-hand panel), and a post-replication component, operating over hours (right-hand panel). The actors identified to take part in each phase are indicated at the bottom. Full red lollipops indicate the methylated 5mC and empty lollipops indicate the unmethylated (unrestored) 5mC.

The Meissner group combined BrdU incorporation with bisulfite treatment and sequencing of newly-synthesized DNA (repli-BS) in human ES cells (134). They reported that, at many sites, nascent DNA becomes methylated significantly later than it is replicated, sometimes by hours. Xu and Corces, also using hES cells, developed a different NGS approach: following EdU incorporation and bisulfite treatment, EdU-containing strands and parental strands were separated and analyzed by sequencing. The methylomes of computationally retrieved pairs of parental and newly-synthesized strands were compared and it was concluded that in most positions nascent DNA strands had regained methylation symmetrically to the parental strands shortly after replication (135), which is discordant with the aforementioned publication. Very recently, another approach was developed in the Zhu lab, which combined EdU incorporation and hairpin ligation of parental and newly-synthesized strands, followed by bisulfite conversion (126). This technique led to similar conclusions as Charlton *et al.* (134), i.e. that a first wave of maintenance methylation, in which 50% of hemi-methylated DNA is converted within 5 min, occurs rapidly and is likely replication-coupled. However, achieving full re-methylation of the genome takes many hours longer than replication; in other words, a slow replication-independent activity also takes place (Figure 3). The early-replicating regions, which were specifically studied in the assay, took up to 6 h to regain 100% of the initial methylation.

Interestingly, the loss of DNA methylation that occurs during aging and in tumors is more prevalent in late-replicating regions, suggesting that incomplete post-replicative methylation may be at fault (136). Statistical analysis of repli-BS data also supports that CpG re-methylation occurs with very different speeds in distinct genomic regions, which correlate with the local density of CpGs. Mathematical modelling revealed that the kinetics of restoration between the neighboring CpG motifs in the genome is correlated (137), suggesting that either DNMT1 is processive through stretches of chromatinized template, or that the recruitment of DNMT1 in nearby loci is coregulated.

Ming *et al.* (126) also reported the effects of key mutations in DNMT1, UHRF1 and other factors. The H170V mutation of DNMT1, which prevents interaction with PCNA, affected the early kinetics of re-methylation, but this delay was entirely compensated by post-replication mechanisms, again confirming that there may be two different phases during methylation maintenance. The same result was obtained with a LIG1K126A mutant, which no longer recruits UHRF1. Inactivating the TTD of UHRF1 (which prevents interaction with H3K9me3 and LIG1K126me3) led to a delay in both the early and the late phases of maintenance but, again, these delays were fully compensated by other mechanisms. Finally, the authors showed that regions bearing H3K9me3 are slower to methylate, but that UHRF1 interaction with H3K9me3 alleviates this intrinsic disadvantage. The kinetics data on the mutants can guide us in the future for placing the different regulatory events relative to each other. We note that, while these data have yielded precious insight, they could be augmented by using new approaches, such as the sequencing of long molecules by Nanopore sequencers.

The fidelity of maintenance has also been a question of interest: how precise is DNMT1 at re-establishing parental methylation patterns? Biochemistry coupled to NGS has recently revealed that DNMT1 activity can be strongly influenced by the sequence context around the target CpG, with the worst sequences being 100-fold less efficiently methylated than optimal sequences (138). Interestingly, the least efficiently methylated sequences are under-represented in the mammalian methylomes, suggesting they have been progressively lost through evolution.

In mouse ES cells, a combination of well-designed KO combinations and careful modeling has also recently shown that DNMT1 is inherently imprecise, yet that its errors are compensated by correction mechanisms (48). The question of fidelity is likely related to kinetics, as regions that are slow to get remethylated may eventually lose the mark. Data in HeLa cells do indeed point in this direction (126). The effect of different DNMT1 and UHRF1 mutations on kinetics has been tested, but their effect on fidelity is not known.

A third concept related to the speed and fidelity is that of the potential disparities between the leading and lagging strand of two newly replicated regions of the genome. As discussed above, the replication fork has two intrinsically distinct arms, so the mechanisms of DNA methylation maintenance could well differ on the leading and lagging strand. In support of this idea, Ming *et al.* found that newly synthesized DNA replicated by the lagging strand re-methylates faster than the one replicated by the leading strand (126). This might have to do with different efficiencies in the recruitment and activation of UHRF1 and DNMT1, the relative speed at which nucleosomes are reassembled, or the inherent sequence skew between the leading and lagging replicated strands (139,140).

It also remains to be determined whether this has functional relevance, for instance in the physiological situations that we will now discuss.

### Section 5. Regulating DNA methylation maintenance in physiological and disease settings

As described above, DNA methylation maintenance is a complex mechanism with many opportunities for regulation. Within a normal cell cycle, the abundance and activity of UHRF1 and DNMT1 are subject to controls that result in higher S-phase abundance (115,116,120). More generally, the activity of these actors can be modulated by post-transcriptional modifications, expression of splicing variants with different domains, and interaction with partners (proteins, nucleic acids, lipids, or possibly other metabolites). In addition, any events that influence DNA replication and chromatin dynamics are also expected to have an effect on DNA methylation maintenance, albeit less directly. An interesting question is how and when these regulatory mechanisms actually come into play, in situations where DNA methylation levels change.

Primordial Germ Cells undergo a profound epigenetic reprogramming as they differentiate from the somatic lineage; this includes a genome-wide demethylation of DNA (141,142). The mechanisms are complex and involve the TET proteins, which are active DNA demethylases (143). However, PGCs divide actively, do not express UHRF1, and do not show recruitment of DNMT1 to replication foci, therefore passive DNA demethylation, by lack of maintenance, probably also contributes to their loss of methylation (144). It is unclear what makes UHRF1 not expressed or unstable in PGCs, and the regulation could be transcriptional, translational, post-translational, or a combination of those. In a different physiological context, T_reg_ cell differentiation, the TGFß signaling cascade was shown to cause UHRF1 degradation, resulting in partial demethylation, transcriptional activation of Foxp3, and the acquisition of a T_reg_ phenotype (145).

Mouse ES cells grown in serum can be reprogrammed to naïve pluripotency by a transfer to 2i medium; this is accompanied by a rapid 4-fold decrease in the global meCpG content (146–148). A decrease in DNA methylation maintenance plays a major role in this (149). It may be driven by decreased abundance of H3K9me2, by removal of UHRF1 from chromatin (150), or by the destabilization of UHRF1 (149), which itself can be targeted for proteosomal degradation by PRAMEL7, which is highly induced in 2i (151).

Oocytes are non-replicating cells, and therefore they have no maintenance methylation. Nevertheless, they express DNMT1 and UHRF1. Early work had shown the existence of an oocyte-specific isoform of DNMT1, which is cytoplasmic in maturing oocytes (152). Recent publications have shown that, in these cells, UHRF1 and DNMT1 are actually responsible for de novo methylation, and that this activity is limited by the factor Dppa3/Stella, which removes UHRF1 from chromatin (153,154). Further studies will hopefully shed light on what conditions promote the maintenance activities of UHRF1 and DNMT1 relative to their de novo activity. Also, oocytes exemplify the existence of splicing variants of DNMT1. Splicing variants of UHRF1 also exist in the mouse and modify the localization and binding behavior of UHRF1 (108), however the physiological role of these variants remains to be discovered.

Finally, some of the actors involved in DNA methylation maintenance are also linked to disease.

UHRF1 is an oncogene, as its overexpression is sufficient to cause hepatocellular carcinoma (155). Paradoxically, UHRF1 overexpression actually causes hypomethylation, possibly because it destabilizes DNMT1 (92), or because it sequesters it in an inactive form. Also, while abnormal maintenance methylation may contribute to tumorigenesis, it seems that inhibition of de novo methyltransferases also plays a role (156). What causes the overexpression of UHRF1 in human cancers is unclear. DNMT1 is also mutated or aberrantly expressed in tumors. In breast cancer stem cells, DNMT1 is increased and is necessary for cell survival (157). However, it is unknown whether the maintenance function of DNMT1 is crucial in this context, or whether maintenance-independent functions are also important.

Besides tumors, DNMT1 is also altered in neurodegenerative diseases. This was first discovered upon exome sequencing in patients with hereditary sensory and autonomic neuropathy type 1 with dementia and hearing loss (HSAN1E) (158). The patients have missense mutations in the RFTS domain of DNMT1. These mutations affect the targeting of DNMT1 (158), its interactions with UHRF1 (159), and correlate with a loss of methylation that is the presumed cause for the pathogenesis. Distinct mutations within the RFTS of DNMT1 are also found in a different neurological condition, ADCA-DN (autosomal dominant cerebellar ataxia, deafness and narcolepsy) (160). The amino acids mutated in HSAN1E and ADCA-DN are physically close (161), so the mechanistic basis for the overlapping yet different syndromes remains to be established.

## DISCUSSION

As we have discussed, complex mechanisms have evolved in mammals to ensure that DNA methylation patterns are faithfully reproduced through cell generations. Among the key actors of this process are the enzymes DNMT1 and UHRF1, which are themselves tightly controlled at the level of their expression, localization, stability, and activity. Besides their interest for basic research, these findings are important to understand human diseases, and may be important for devising new therapies. This is especially true in the context of cancer, where DNA methylation appears as a key vulnerability of tumor cells, a lock that when lifted can induce cell-intrinsic toxic effects (162), as well as a greater responsiveness to immunotherapy (163); DNMT1, UHRF1 and both in combination are promising targets in this context (164).

We have shown clear illustrations of how the methylation maintenance machinery relies on DNA and chromatin replication factors: DNMT1 interacts with PCNA and UHRF1 with LIG1, for example. An open question is whether, conversely, the replication machinery requires the DNA methylation apparatus for optimal function (in those organisms that have DNA methylation, of course). An argument against this idea is that mouse and human ES cells genetically engineered to be devoid of DNA methylation show no obvious replication defects. However, it is also clear that DNA hypomethylation in cancer correlates with increased genome instability. This is likely due in part to transcriptional deregulation, but a direct effect of a subfunctional DNA methylation machinery on DNA replication is difficult to rule out.

A situation we have not discussed is that of DNA repair. When a DNA break is repaired by homologous recombination, a long tract of hemi-methylated DNA is generated, and it must be converted back to fully methylated DNA (165). It is unknown if the same events govern replication-coupled DNA methylation maintenance, and repair-coupled maintenance.

To sharpen the focus of this review, we have only discussed DNMT1- and UHRF1-dependent DNA methylation maintenance in the CpG context. However, it is clear that DNMT3A and DNMT3B can also participate to DNA methylation maintenance, especially in cells that express the enzymes at high levels such as stem cells (166,167).

An area where progress has been made is in our understanding of the kinetics of DNA methylation maintenance, and the discovery of rapid, replication-coupled events, but also much slower, post-replication events. Whether and how the latter are linked to the slow phases of chromatin maturation remains to be determined. These kinetic studies will undoubtedly benefit in the future from additional approaches, such as single-molecule, real-time sequencing. This, together with increasing molecular knowledge of the key actors, will allow the community in the years to come to reach a more detailed, complete and realistic picture of the processes of DNA methylation maintenance, how they operate in healthy cells, how they are regulated during developmental transitions, and how their dysfunctions associate with diseases.
